# Full Breastfeeding and Obesity in Children: A Prospective Study from Birth to 6 Years

**DOI:** 10.1089/chi.2017.0335

**Published:** 2018-07-01

**Authors:** Juan Antonio Ortega-García, Nicole Kloosterman, Lizbeth Alvarez, Esther Tobarra-Sánchez, Alberto Cárceles-Álvarez, Rebeca Pastor-Valero, Fernando Antonio López-Hernández, Manuel Sánchez-Solis, Luz Claudio

**Affiliations:** ^1^ Pediatric Environmental Health Speciality Unit, Laboratory of Environmental and Human Health (A5), Department of Paediatrics, Institute of Biomedical Research, IMIB-Arrixaca, Virgen de la Arrixaca University Hospital, University of Murcia, Murcia, Spain.; ^2^ Departamento de Métodos Cuantitativos e Informáticos. Universidad Politécnica de Cartagena, Cartagena, Spain.; ^3^ Division of International Health, Mount Sinai School of Medicine, New York, NY.

**Keywords:** breastfeeding, childhood obesity, childhood overweight, cohort study, Spain

## Abstract

***Background:*** Obesity is a major public health crisis among both children and adults and contributes to significant physical, psychological, and economic burden. We aim to investigate the effect of duration of breastfeeding on excessive weight and obesity at 6 years of age.

***Subjects/Methods:*** Data on breastfeeding and child anthropometric measurements were collected in a birth-cohort study in Murcia, Spain (*n* = 350). Breastfeeding status and body mass index (BMI) were established according to WHO definitions. Other factors potentially related to children's weight were considered. Multiple log-linear and ordinal regressions were used to analyze the effects of breastfeeding on overweight and obesity when considering potential confounders.

***Results:*** 33% and 17.3% of children in the study were of excess weight and obesity, respectively. Univariate predictors of BMI in children aged 6 were as follows: pregestational maternal BMI (kg/m^2^) (*R*^2^ = 0.127, *p* < 0.01); full breastfeeding (weeks) *R*^2^ = −0.035, *p* < 0.01); infant weight gain (kg) (*R*^2^ = 0.348, *p* < 0.01); and maternal alcohol consumption during pregnancy (g/day) (*R*^2^ = 0.266, *p* < 0.01) at age 6. In the ordinal logistic regression, full breastfeeding was associated with a significant decrease in obesity −0.052 (95% CI, −0.10 to −0.003).

***Conclusions:*** The delay of bottle feeding introduction may have a protective effect against obesity at 6 years of age. Our findings reinforce the need for greater support of breastfeeding and to promote a healthy environment and antipoverty interventions during pregnancy and infancy, alongside other strategies for obesity prevention.

## Introduction

Obesity is a major public health crisis among both children and adults and contributes to significant physical, psychological, and economic burden.^1^ The prevalence of childhood obesity is increasing in both low- and high-income countries.^2,3^ In 2010, around one in three children in the EU aged 6–9 years were overweight or obese and rates have been increasing since.^4^ In Spain from 2011 to 2012, the prevalence of childhood excess weight and obesity was 29.7% and 9%, respectively.^5^ Obesity at an early age often continues into adulthood and confers a major risk for insulin resistance, impaired glucose tolerance, hypertension, dyslipidemia, cardiovascular disease, and cancer.^6–9^

A mounting body of evidence suggests that breastfeeding may also play a role in programming noncommunicable disease risk later in life. Evidence in the literature analyzing the protective effects of breastfeeding against childhood adiposity has yielded controversial conclusions. Some studies have found no significant correlation between breastfeeding and different measures of overweight.^10,11^ However, other authors have found that children who had not been breastfed^12,13^ or who were breastfed for a shorter period of time^14,15^ showed increased risk of overweight and obesity. Table 1^6–8,10–24^ describes various cohort studies on this topic. Identifying modifiable determinants of childhood obesity, such as breastfeeding, are critical for developing effective intervention strategies for this chronic disease.

**Table 1.** Cohort Studies Analyzing Breastfeeding and Other Factors on Obesity^6–8,10–24^

Author	Country	Sample size	Study design	Age at measurement	Comparison groups	Outcomes (reference)	OR (CI 95%)
O'Callaghan et al.^10^	Australia	2034	PC	5 years	Ever breastfed vs. never breastfed	Obesity BMI </ = 94th percentile	0.71 (0.43–1.25)
Armstrong et al.^13^	Scottland	32,200	RC	39–42 months	Exclusively breastfed vs. bottle fed at 6–8 months	Obesity (BMI </ = 95th percentile)	0.72 (0.65–0.79)
Bergmann et al.^12^	Germany	1314	PC	6 years	Breastfed for ≥3 months vs. Breastfed for <3 months	Obesity (BMI >97th percentile	0.46 (0.23–0.92)
Grummer-Strawn et al.^8^	US	12,587	RC	4 years	Breastfed for ≥12 months vs. Never breastfed	Overweight or Obesity (CDC)	0.72 (0.65–0.80)
Burke et al.^16^	Australia	1672	PC	1–8 years	Breastfed for ≥12 months vs. Never breastfed	Obesity (CDC)	1.83 (1.20–2.78)
Dubois et al.^17^	Canada	2103	PC	4.5 years	Breastfed for >/ = 3 months vs. breastfed for <3 months	Obesity (CDC)	1.0 (0.7–1.5)
Scholtens et al.^18^	Netherlands	2043	PC	8 years	Breastfed for >16 weeks vs. Breastfed for ≤16 weeks	Overweight or Obesity (IOTF)	0.70 (0.27–1.74)
Huus et al.^11^	Sweeden	14,244	PC	5 years	Exclusively breastfed for ≥4 months vs. Exclusively breastfed for <4 months	Obesity (WHO)	0.82 (0.55–1.23)
Kwok et al.^19^	China	7026	PC	7 years	Exclusively breastfed for ≥3 months vs. Never breastfed	Overweight or Obesity (IOTF)	1.09 (0.83–1.43)
Shields et al.^20^	Australia	2533	PC	21 years	Breastfed for >4 months vs. Never breastfed	Obesity (WHO)	0.93 (0.63–1.39)
Van Rossem et al.^21^	US	1579	PC	3 years	Exclusively breastfed vs. Mixed vs. bottle fed at 6 months	Obesity (IOTF)	.98 (.87–1.11)
McCrory et al.^22^	Ireland	3177	RC	9 years	Breastfed for ≥26 weeks vs. Never breastfed	Overweight and Obesity (IOTF)	0.62 (0.39–0.99)
Jwa et al.^6^	Japan	41,572	PC	5.5, 7 and 8 years	Exclusively breastfed vs. Mixed vs. bottle fed at 6 months	Overweight and Obesity (IOTF)	0.68 (0.53–0.87)
Shi et al.^7^	Canada	968	PC	6–11 years	Exclusive vs. Not exclusive vs. No breastfeeding at 6 months	Overweight and Obesity (WHO)	0.44 (0.31–0.63)
Yamakawa et al.^14^	Japan	30,780	PC	7 and 8 years	Partial vs. exclusive breastfeeding at 6–7 months	Overweight and obesity (IOTF)	0.85 (0.69–1.05)
Moss et al.^23^	US	14,150	PC	2 and 4 years	Breastfed vs. not breastfed measured at 9 months	Obesity (CDC)	0.64 (0.51–0.80)
Wallby et al.^15^	Sweeden	30,508	PC	4 years	Breastfed for ≥12 months vs. Never breastfed	Obesity (WHO)	0.55 (0.34–0.90)
Wang et al.^24^	US	1234	PC	5 years	Breastfed for ≥6 months vs. Never breastfed	Overweight and Obesity (CDC)	0.58 (0.36–0.94)

BMI, body mass index; IOTF, international obesity task force; RC, retrospective cohort; PC, prospective cohort; OR, odds ratio.

The mechanisms underlying the association between breastfeeding and obesity highlight three protective effects which may lead to lower body fat levels in breastfed infants. Breastfeeding helps encourage self-regulation of intake, reduce interference of caregivers in creating positive feeding behaviors, and providing necessary chemical components to regulate energy metabolism.^25^ Human milk contains hormones that moderate energy metabolism and food intake. Various hormones, including leptin, insulin, adiponectin, and obestatin, can activate various pathways that regulate hunger, depending on energy requirements, possibly also via epigenetic processes.^26,27^Also, the beneficial effects of breastfeeding on obesity could be mediated partly by programming a healthier composition of gut microbiome, inducted by some breast milk components (nondigestible oligosaccharides).^28^ Differences in hormone and protein content between breast milk and formula may play a role in increasing risk of excess weight and obesity.^26^ Also, recommendations have been made to study mode of administration and its impact on childhood obesity to determine its role in appetite regulation regardless of substance consumed.^15^

This article uses the MALAMA (Medio Ambiente y Lactancia Materna) longitudinal population-based cohort to analyze the relationship between breastfeeding and childhood outcomes.^29,30^ In this study, we examined the relationship between the duration of breastfeeding and body mass index (BMI) at 6 years of age in a Mediterranean Region, accounting for other factors that influence obesity.

## Materials and Methods

### Study Participants

Murcia is a European region located in southeast Spain, with a total population of 1,472,000 inhabitants (259,083 < 15 years) in 2013.^31^ The study was conducted within four health areas (1, 6, 7, and 9) whose reference maternity hospital is the Clinical University Hospital “Virgen de la Arrixaca” with a reference population of 747,233 persons and 8150 newborns per year.

MALAMA is an ongoing longitudinal, prospective cohort study from birth until 18 years of age that examines the relationship between breastfeeding duration and childhood development. The MALAMA project follows 430 mother–child pairs, from two population-based birth cohorts.^29,30,32^ This study was based on the second *de novo* MALAMA cohort, where 350 mother–child pairs were randomly selected one out of two after giving birth at Clinical University Hospital “Virgen de la Arrixaca” between June 10 and July 20, 2009.^30,32^ The central location of the maternity hospital facilitates ease of access to cohort for follow-up for all newborns and family in the study. The MALAMA project was approved by the Ethics Committee and the Institutional Review Board of the Clinical University Hospital “Virgen de la Arrixaca.”

The participants included in this study were healthy newborns born full-term (>37 weeks of gestation), weighing >2500 g at the study hospital, first born, and with Apgar test given at 1 minute and 5 minutes with a minimum score of 7 and 8, respectively. Participants were excluded from the study if a telephone number was unavailable to contact the parents, newborns were admitted to the neonatal unit during the first 48 hours, and a linguistic barrier was present that was unable to be overcome either due to the lack of an available interpreter or the inability to hold a conversation.

Recruitment and the first interview were conducted face-to-face with either the mother or both parents present at the time of neonatal discharge. In addition, face-to-face interviews were conducted at both the first month and the 24th month. They were interviewed by a nurse trained in breastfeeding and research methodology, utilizing a carefully developed questionnaire known as “la hoja verde” or the “green page” (GP). GP on reproductive environmental health includes the standard clinical record of pregnant or lactant women and constitutes a series of concise and basic question through which the healthcare professional identifies environmental exposure during these periods.^33–35^ Follow-up was done through a series of phone calls at 1, 3, 6, and 12 months. Up to five phone calls were placed to establish contact with the study participants before lost to follow-up. A scheduled well-child care physical examination includes anthropometric measures at 1, 2, 4, and 6 years old. The anthropometric measurements of children at 6 years were obtained from a growth monitoring program within the pediatric primary care unit from the child ambulatory history. Well-child visit programs are an important tool utilized by healthcare providers to screen for medical and developmental issues.^36^

From the 350 mother–newborn dyads randomly recruited, 15 did not meet the inclusion criteria and 327 dyads (97.6%) agreed to participate in the study. There were three couples (1%) who were lost to follow-up at 1 year and 1 (0.3%) that abandoned the study at 1 year. Of the remaining children, 324 provided information regarding full breastfeeding, and information regarding BMI at 6-year mark was available in 231 (71.3%) children for the study.

### Infant Feeding Practice

Data were collected on breastfeeding, as defined by the World Health Organization (WHO) recommendations. “Exclusive breastfeeding” (EBF) means that the infant receives only breast milk, no other liquids or solids are given, and “Full breastfeeding” (FBF) includes exclusive (no other liquid or solid is given to the infant) and almost exclusive (vitamins, mineral water, juice, or ritualistic feeds are given infrequently in addition to breastfeeds or non-nutritive foods).^37^ The duration of full breastfeeding was noted until the date bottle-feeding was first introduced. Any Breastfeeding (ABF) is the duration of lactation. The analysis of breastfeeding duration was used as a continuous quantitative variable measured as days that mother spent: EBF, FBF, and ABF.

### Child's Overweight and Obesity Status

Anthropometric measurements, obtained from well-child examinations, included weight and height. To weigh and measure children, standardized measurement procedures were used with the following equipments: <2 years: baby scale SECA 717 (to the nearest 2 g) with measuring rod 231 (to the nearest 1 mm) and >2 years: flat scale SECA 872 (to the nearest 50 g) and mobile stadiometer SECA 217 (to the nearest 1 mm).

BMI was calculated using the following formula: weight (kg)/height (m)^2^. Childhood excess weight and obesity was defined using child growth standards established by the WHO. Using this measure, childhood excess weight is defined as BMI > one standard deviation body mass index (BMI) for age and sex, overweight is defined as values between 1 and 2 standard deviations BMI for age and sex, and obesity defined as BMI > two standard deviations BMI for age and sex.^38^

### Covariates

The following sociodemographic and exposure factors studied were obtained from GP: sex, birth weight, weight gain in first year of life, maternal age, pregestational maternal BMI, mother's alcohol consumption during pregnancy (during 2nd–3rd trimester), and smoking during early pregnancy and 1-year postpartum. In addition, nationality (native/foreign), parental education level (no education-primary/secondary/university), family income in euros (€) per month (<800/800–1500 €/1501–2500 €/>2500 €), and maternal employment type during periconceptional period were studied.

### Statistical Analysis

The data analysis was computed utilizing the Statistical Package for the Social Sciences version 21(SPSS, Chicago, IL)^39^ and the mgcv R package. First, univariate analyses were performed. To obtain predictor variables, the comparisons of all variables with excess weight and obesity were made using Chi-squared tests, ANOVA test, Pearson's correlation, and Spearman's rho correlation. Significant results are reported alongside descriptive statistics in Table 2. A log-linear regression analysis was performed, in which the outcome variable was BMI at 6 years old. Analyses included variables that were significantly (*p* < 0.05) associated with excess weight or obesity in the univariate analyses at age 6. We use Generalized Additive Models (GAMs) to identify complex nonlinear relationships between the response and explanatory variables.^40^ Using results from the GAM model, we used an ordinal logistic regression to model the nonlinear relationship between variables. For both the log-linear regression and ordinal logistic regressions, we utilized 192 participants for whom we had responses for all variables and anthropomorphic measurements. We found no statistically significant differences in socioeconomic status (SES), “Full breastfeeding” and mother pregestational BMI between this group and those lost during the follow-up. Effects were considered statistically significant with *p*-value <0.05 and ORs with a 95% CI that did not include 1.

**Table 2.** Descriptive Statistics of Study Sample

Variable	n	N (%)	Mean (CI 95%)	Correlations^a^, p-value	ANOVA t-test	RR (CI 95%) univariate
Child BMI at 6 years	231	n.a.	16.36 (16.06–16.67)	n.a.	n.a.	n.a.
Obesity status at 6 years						
Normal weight	156	(67.5)	n.a.	n.a.	n.a.	n.a.
Overweight	35	(15.2)	n.a.	n.a.	n.a.	n.a.
Obese	40	(17.3)	n.a.	n.a.	n.a.	n.a.
EBF (weeks)^b^	324	n.a.	7.56 (6.55–8.58)	−0.16, 0.01	n.a.	−0.04 (−0.07 to −0.01)
FBF (weeks)^b^	324	n.a.	11.64 (10.45–12.84)	−0.17, <0.01	n.a.	−0.04 (−0.06 to −0.01)
ABF (weeks)	323	n.a.	27.96 (25.45–30.46)	0.02, 0.76	n.a.	
Sex of child	324				0.24	
Male		177 (54.7)	n.a.	n.a.		n.a.
Female		147 (45.3)	n.a.	n.a.		n.a.
Birth weight (g)	324	n.a.	3270 (3230–3320)	0.10, 0.14	n.a.	n.a.
Weight gain first year (kg)^b^	287	n.a.	6.86 (6.71–7.01)	0.26, 0.01	n.a.	0.48 (0.24–0.72)
Maternal origin	324				0.52	
Native born		262 (81)	n.a.	n.a.		n.a.
Foreign born		62 (19)	n.a.	n.a.		n.a.
Maternal age (y)	324	n.a.	31.57 (31.00–32.14)	−0.06, 0.34	n.a.	n.a.
Maternal Pre-Gestational BMI^b^	219	n.a.	24.59 (23.96–25.23)	0.26, <0.01	n.a.	0.48 (0.24–0.72)
Maternal smoking						
Periconceptional^b^	324					
Smoking		204 (63.0)	n.a.	n.a.	0.03	0.69 (0.06–1.32)
Not Smoking		120 (37.0)	n.a.	n.a.	n.a.	n.a.
Cigarettes/week		n.a.	27.90 (22.66–33.13)	0.20, <0.01	n.a.	0.01 (0.00–0.02)
Postnatal (1 year)^b^	323					
Smoking		84 (26.0)	n.a.	n.a.	0.01	0.88 (0.19–1.58)
Not smoking		239 (74.0)	n.a.	n.a.	n.a.	n.a.
Cigarettes/week		n.a.	12.79 (9.55–16.04)	−0.06, 0.67	n.a.	n.a.
Maternal alcohol intake (Pregnancy)^b^	324				0.16	
Yes		37 (11.4)	5.09 (3.24–6.94)	n.a.		n.a.
No		287 (88.6)		n.a.		n.a.
Alcohol (Grams/Day)		n.a.		0.23, <0.01		0.21 (0.09–0.32)
Maternal Occupation	324				0,15	
outside the home		158 (48.9)	n.a.	n.a.		n.a.
at home		165 (51.1)	n.a.	n.a.		n.a.
Education level: Mother^b^	324	70 (21.6)	n.a.	−0.17, 0.01	0.05	Ref
None/Primary		154 (47.5)	n.a.			−0.13 (−0.93 to
Secondary			n.a.			0.67)
University		100 (30.9)				−0.86 (−1.75 to −0.02)
Education level: Father^b^	324			−0.19, <0.01	0.04	
None/primary		93 (29.8)	n.a.			Ref
Secondary		144 (46.2)	n.a.			−0.37 (−1.01 to 0.34)
University		75 (24.0)	n.a.			−1.08 (−1.96 to −0.24)
Family net income (€/month)^b^	323			−0.26, <0.01	<0.01	
<800		38 (11.8)	n.a.			1.64 (0.46–2.81)
800–1499		124 (38.4)	n.a.			1.31 (0.50–2.13)
1500–2500		93 (28.8)	n.a.			0.61 (−0.22 to 1.44)
>2500		68 (21.1)	n.a.			Ref

^a^ Correlation Pearson coefficient between “Child BMI at 6 years” and the correspondent variable.

^b^ *p* < 0.05 for excess weight and obesity at 6 years old in univariate analysis. Ref = Category used as reference for RR. Univariate statistics used include Chi-Squared tests, Pearson's Correlation, Spearman's Correlation, ANOVA, *t*-test to obtain predictor variables.

ABF, any breastfeeding; EBF, exclusive breastfeeding; FBF, full breastfeeding; n.a., not applicable; RR, relative risk.

## Results

The median duration of FBF was 63.5 days and 21% of children were FBF at least 6 months. The prevalence of ABF at 12 months was 19.2%. At 6 years of age, 32.8% and 17.7% of children were categorized as being of excess weight and obesity, respectively. Descriptive statistics of sociodemographic variables are shown in Table 2. Children who had high weight gain in their first year of life and whose mothers who had higher BMI, smoked, or drank alcohol during pregnancy, parents with low educational attainment were more likely to be of excess weight than those who didn't have these factors. Children who were exclusive or full breastfed were less likely to be of excess weight or obese at this age in the univariate analysis.

Predictors variables of BMI in children aged 6 years by log-linear regression are shown in Table 3. Pregestational maternal log BMI (kg/m^2^) (*R*^2^ = 0.127, *p* < 0.01); full breastfeeding (weeks) *R*^2^ = −0.035, *p* < 0.01); infant weight gain (kg) (*R*^2^ = 0.348, *p* < 0.01); and maternal alcohol consumption during pregnancy (g/day) (*R*^2^ = 0.266, *p* < 0.01) were found to be predictive of excess weight at age 6.

**Table 3.** Log-Linear Regression of Body Mass Index with Predictor Variables in Children Aged 6 Years

Predictor variable	Regression coefficient	95% confidence interval	p value
Exclusive BF (weeks)	0.021	−0.028 to 0.070	0.44
FBF (weeks)	−0.035	−0.065 to −0.006	0.01
Any BF (weeks)	0.007	−0.007 to 0.028	0.28
Maternal BMI (kg/m^2^)	0.127	0.058–0.197	<0.01
Infant weight gain (kg)	0.348	0.072–0.624	0.01
Alcohol consumption during pregnancy (g/d)	0.266	0.123–0.408	<0.01
Mother smoking during periconceptional (cig/w)	0.004	−0.005 to 0.013	0.41
Mother smoking (dichotomous)	−0.191	−1.314 to 0.933	0.74
Mother education	−0.004	−0.604 to 0.596	0.99
Father education	0.186	−0.368 to 0.739	0.51
Net income	−0.396	−0.860 to 0.068	0.09

End Point: BMI (6 years) *r*^2^ = 0.261, Durbin–Watson = 2.15.

The following predictor variables were included: Maternal BMI, Infant weight gain (kg), mother smoking during periconceptional (dichotomous and cig/week) periods, Exclusive BF, ABF and FBF, mother and father educational level (ordinal), net income per month (ordinal), Infant weight gain and alcohol intake during pregnancy (g/day). To obtain predictor variables the comparisons of all variables were made using the unpaired Student's *t* test, ANOVA test and Pearson/Spearman's rho correlation.

**FIG. 1.**
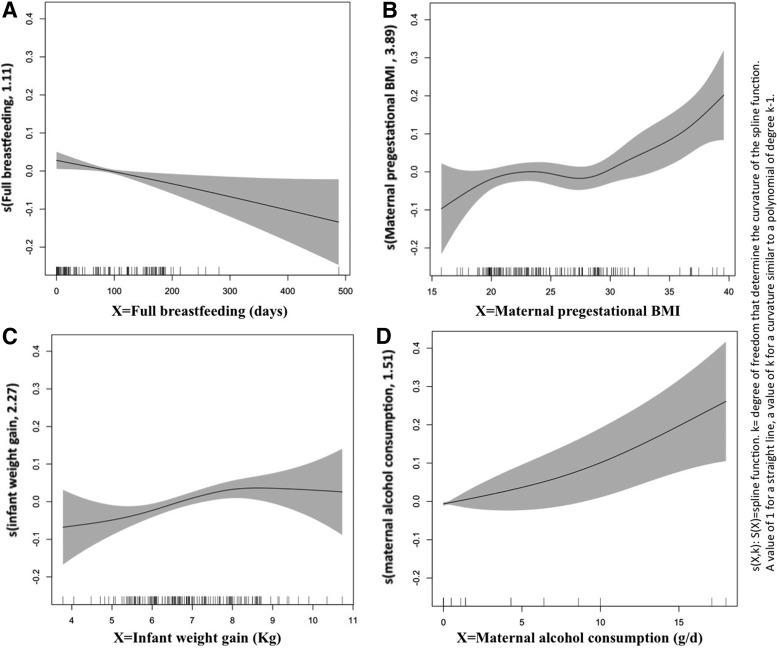
GAMs to identify the form of the functional relationship between the log BMI at 6 years and the explanatory variables. **(A)** There is a linear relationship with full breastfeeding (*r* = 1); There is a nonlinear relationship (*r* >/ = 2) with **(B)** maternal pregestational BMI **(C)** infant weight gain and **(D)** maternal alcohol consumption. BMI, body mass index; GAM, generalized additive model.

Figure 1 shows results from GAM model that analyzed linear trends or functional relationship between log BMI and four variables. Maternal pregestational BMI, infant weight gain, and maternal consumption were found to have a nonlinear relationship (*r* or the smooth term >2). Because of this a multivariate ordinal logistic analysis was conducted and results can be seen in Table 4. An increase in full breastfeeding (expressed in weeks) was associated with a decrease in overweight/obese of −0.052 (95% CI, −0.10 to −0.003). Maternal BMI and weight gain in the first year of life were also associated with an increase in overweight/obese of 0.093 (95% CI, 0.023 to −0.163) and .407 (95% CI, 0.172 to −0.642), respectively. Family income and parental education were not statistically significant in this model. However, it is observed an inverse relationship between level of income at birth and average BMI at 6 years old. In our study, 12% of children belong to families living in relative poverty in 2009; and the proportion of obese is significantly higher among the poor (33.3%) than rich individuals (11.5%). While not significant, we observed a growing risk of high BMI as income declines.

**Table 4.** Ordinal Regression: Risk Factors Associated with Overweight/Obesity at 6 Years

				CI 95%	
Variable	Wald	Significance	Regression coefficient	Min	Max
EBF (weeks)	0.912	0.34	0.027	−0.29	0.083
FBF (weeks)^a^	4.151	0.04	−0.052	−0.10	−0.003
ABF (weeks)	2.626	0.11	0.015	−0.003	0.032
Maternal BMI (Kg/m^2^)	6.791	<0.01	0.093	0.023	0.163
Weight gain first year of life (g)^a^	5.731	0.01	0.407	0.172	0.642
Alcohol intake during pregnancy (g/d)	3.637	0.05	0.136	0.024	0.236
Periconceptional maternal smoking (cig/week)	0.001	0.97	0.003	−0.003	0.009
Monthly net income <800 €	1.592	0.17	1.173	−0.594	2.739
800–1499 €	1.149	0.28	0.575	−0.597	2.039
1500–2500 €	0.383	0.53	0.051	−0.881	1.560
Maternal University Education (Yes/No)	0.134	0.71	−0.160	−1.020	0.700
Paternal University Education (Yes/No)	0.007	0.93	−0.036	−0.907	0.835

^a^ *p* < 0.05.

## Discussion

Our study shows that childhood obesity is a significant public health concern in the Region of Murcia: 1/3 of children had excess weight, similar to other results in this region.^41^ We observed a small, yet statistically significant, protective effect of FBF on obesity in 6-year-old children. A reduction of 3.5% of BMI in 6-year-old children by each week increase of FBF was observed, while the other variables in the model are held constant. More complex is the interpretation of ordinal regression model coefficients. Case of FBF, one week increase in FBF resulted in a 5.2% decrease in the ordered log-odds of being in a higher BMI category, while the other variables in the model are held constant. The protective dose–response effect of breastfeeding on overweight or obesity is observed even for relatively short periods of breastfeeding. In addition, maternal BMI, children's weight gain in the first year of life, and exposure to alcohol and poverty increase the risk of excess weight and/or obesity later in childhood.

The benefits of breastfeeding on both child and maternal health are well known. In US Surgeon General's Call to Action to support breastfeeding, it is noted that late weaning is associated with a protective effect in children against infections, eczema, hospitalization, SIDS, and chronic diseases such as leukemia, type 2 diabetes, asthma, and obesity.^42^ The WHO also found significant association between the duration of breastfeeding and type 2 diabetes, cholesterol, and performance in intelligence tests.^43^ In their analysis, they suggest that there is a small reduction in prevalence in childhood weight gain (10%) in children exposed to longer durations of breastfeeding, but warn about lack of adjustments for confounding factors which may attribute to this effect.^43^ Research conducted throughout various countries have yielded inconclusive results regarding the protective effects of breastfeeding on childhood adiposity.^6–8,10–24^

In the study, maternal BMI was significantly associated with BMI, overweight and obesity in 6-year-old children. Our findings are consistent with previous literature that identifies maternal obesity status as an important factor in childhood^11,15,17^ and adulthood^20^ BMI. Mechanisms explaining this relationship include inheritance of genes that make child susceptible to excess weight, mother's role in shaping eating habits and activity environment, and the effects of maternal obesity as a fetal modulating environmental factor during pregnancy.^44,45^ Intrauterine environment can alter metabolism through changes in gene expression.^46^

Our results found that prolonging the introduction of formula feeding decreased the risk of excessive weight and obesity. We utilized variables that have been standardized by the WHO for both obesity and breastfeeding. We observed significant results with FBF, a variable that is a more realistic measure than exclusive breastfeeding, which is more demanding and difficult to get practice by definition. However, we did not observe a similar significant effect with ABF. The use of a standardized definition for breastfeeding is critical to evaluate the relationship between breastfeeding and obesity on an international scale. While the effect of 1 day of FBF was small, it was significant in providing an immediate and accumulative protective effect. The effect of breastfeeding on obesity has been studied in cohorts ranging from 2 years^23^ to 21 years,^20^ and a protective effect has been observed up to 6–11 years.^7^ Most of the studies analyzing breastfeeding and obesity utilize dichotomous variables.^7,12,13,17,18,21,23,24,47,48^ Our results are consistent with multiple studies that have found significant inverse associations between breastfeeding type and duration and child's weight status.^6,8,14–16,22^ However, some studies have found nonsignificant associations between breastfeeding and childhood overweight.^11,19,20^ These differences in results may be attributed to cultural differences in the population analyzed, differences in definitions of breastfeeding, obesity, and covariates, and also differences in age at which BMI was measured.

In our study, periconceptional and postnatal smoking was significantly associated with excess weight in the univariate analysis. Exposure to smoking during pregnancy has been associated with childhood overweight and correlated with child obesity, although the biological mechanism for this epidemiological link is not fully understood.^15,17,49^ Maternal smoking is related to low birth weight, which is associated with catch-up growth early in life, which is associated with overweight and obesity in childhood.^12,17^ Mothers who smoke during pregnancy are more likely to be less educated and not breastfeed than nonsmoking mothers.^11,14,29,30,49^ Exposure to nicotine *in utero* has been associated with increased body fat and weight.^50^ Maternal smoking in pregnancy has also been suggested to affect the appetite regulation system in the developing brain, making it a possible independent risk factor for overweight in children and can be a proxy for other environmental factors present during postnatal development such as diet and physical activity.^49^ We also found a significant association between increased maternal alcohol consumption during pregnancy and increased childhood BMI. While maternal smoking during pregnancy is studied as a risk factor for child obesity, the effects of alcohol consumption on weight outcomes are not as scrutinized.^47,49^ Evidence is available showing that children with partial fetal alcohol syndrome experience higher overweight and obesity rates.^51^ Animal studies, demonstrate that prenatal alcohol exposure leads to insulin resistance and leads to glucose intolerance.^52^ Similar to our study, it is important for future studies to evaluate the interaction between smoking and alcohol consumption for better understanding of their impact on childhood obesity.

Also, our findings showed an association between weight gain in the first year and childhood BMI and excess weight at 6 years old. In a systematic review that analyzed rapid infancy weight gain and subsequent obesity, 21 studies reported a significant positive association.^53^ DuBois' cohort study found that weight gain in the first 5 months was associated with overweight at 4.5 years.^17^ Although there is noted effect of early infant weight gain on childhood BMI, results were not as consistent with other measures of adiposity such as skinfold thickness.^21^ Rapid weight gain in the first two years of life has been linked to obesity, particularly in infants with low birth weight and size (Perng). Although weight gain in the first year of life as part of “catch-up growth” is associated with adverse metabolic effects, there may also be certain benefits to this type of growth in certain groups.^54^ The mechanism explaining how early infant weight gain influences weight status later on in life is unclear. However, it is well understood that early development not only is extremely susceptible to environmental influences but also is influential in later health outcomes.^55^ Infant weight change in the first 6 months of life is associated with both breastfeeding and childhood BMI.^21^ One possible interaction between breastfeeding and weight gain in the first year of life can be explained by differing levels of protein content with breastfeeding and formula feeding.^56^ In a study conducted in five European countries, they found that high protein intake induced increased weight gain velocity during the first months of life, resulting in increased body fat deposition.^57^

Although we observed in the univariate analysis a relationship of parental education levels on childhood overweight and obesity, the effect was not seen in the multivariable regressions. Previous studies have analyzed the effects of parental education and its effect on obesity at different ages. While maternal education was found to have a significant effect on childhood obesity,^12,14,16,20,21^ fewer studies considered paternal education.^6^ Occupational status was also analyzed in previous studies, but differed in the way it was measured.^12,19^ In a Swedish study, they found that maternal employment for less than 3 months during pregnancy was associated with short-term breastfeeding.^11^ Our study assessed the activity level associated with maternal occupation, but did not find a difference between mother's occupation and risk of childhood excess weight and obesity at 6 years old.

We found the economic level of the families to be associated with BMI in children but only in the univariate analysis. Socioeconomic disparities are a considerable risk factor for obesity and the abandonment of breastfeeding.^31^ Children of low SES are more likely to be obese than high-SES children and their rates of obesity are increasing at a much faster rate.^58^ This is particularly important to analyze in Murcia, which is the region with the 5th lowest GDP per capita in Spain.^59^

Extensive information gathered regarding breastfeeding during infancy, before outcomes were measured, allows us to have detailed information about child's feeding habits. Multiple follow-up sessions took place both over the phone and face-to-face about breastfeeding habits, particularly in the first two years of life. By treating each type of breastfeeding and its duration in a continuous manner, using days as unit of measurement, we have data on the exact timing that infants were introduced to bottle feeding at home. The careful and exhaustive data collection on BF minimizes the likelihood of recall bias in the study.

Several limitations must be considered in our study. First, the sample size was limited compared with that of previous studies. Secondly, it is important to consider both recall and selection bias. We have attempted to compensate these limitations by contacting participants with increased frequency to retrieve more accurate information and decrease bias. Our extensive data collection provided us with results that are largely consistent with the rest of the literature. Attrition during follow-up created loss of growth data and maternal BMI limiting the number of participants with completed records in parts of the analysis. Previous studies have noted similar limitations regarding loss of data and high attrition rates in longitudinal studies.^14,15,20^ Finally, a common limitation of observational studies is the inability to adjust for all confounding variables.^6,8,13,14,19,21^

However, the covariables discussed in this study are representative of some of the major underlying risk factors for obesity considered in previous studies. There are some factors, such as heredity and lifestyle factors (diet, physical activity, time spent watching TV/playing computer games/sleep), that were not analyzed in this study. In future studies, we will incorporate some of these variables. Besides, we will conduct a developmental assessment of these children at 8 and 12 years old.

Analysis of overweight and obesity could have been improved by using other effective methods to measure adiposity along with BMI. Both X-ray absorptiometry^21^ and skinfold thickness^12,21,49^ have been suggested and used to better analyze the relationship between breastfeeding and obesity. Nevertheless, BMI is still considered an inexpensive and noninvasive way to measure body fat that is internationally accepted.^9,60^

## Conclusions

In our study, we established a dose-dependent relationship between FBF duration and weight status in early childhood. The early introduction of bottle feeding increased risk for childhood excess weight and obesity. The use of standardized measures, particularly of breastfeeding, will go a long way in better understanding this protective effect internationally. While evidence is still being gathered on this topic, prevention programs against childhood obesity should promote prolonged breastfeeding, the creation of healthier environments during pregnancy, and infancy free of tobacco and alcohol and consider antipoverty interventions.
